# ABO Blood Group Differentials on Survival in Hepatocellular Carcinoma Patients Treated with Chemoembolization

**DOI:** 10.31557/APJCP.2021.22.11.3685

**Published:** 2021-11

**Authors:** Kittipitch Bannangkoon, Keerati Hongsaku, Pimsiri Sripongpun, Apichat Kaewdech, Naichaya Chamroonkul, Teeravut Tubtawee, Teerha Piratvisuth

**Affiliations:** 1 *Department of Radiology, Faculty of Medicine, Prince of Songkla University, Hat Yai, Songkhla, Thailand. *; 2 *Gastroenterology and Hepatology, Faculty of Medicine, Prince of Songkla University, Hat Yai, Songkhla, Thailand. *; 3 *NKC Institute of Gastroenterology and Hepatology, Faculty of Medicine, Prince of Songkla University, Hat Yai, Songkhla, Thailand. *

**Keywords:** ABO, blood group, hepatocellular carcinoma, TACE, chemoembolization

## Abstract

**Background:**

The association between ABO blood group and the prognosis of hepatocellular carcinoma (HCC) remains unclear. We investigated the impact of ABO blood groups as a prognostic factor in HCC patients treated with transarterial chemoembolization (TACE).

**Materials and methods:**

We revisited records of all HCC patients who underwent TACE between January 2007 and December 2019 at a tertiary care hospital. The inclusion criteria were HCC patients, Child-Pugh score A5-B7, and treated with TACE monotherapy. The baseline characteristics of each patient were compared against their blood group and the survival analysis was carried out using Cox’s regression. With Bonferroni adjustment for multiple comparisons, P-values <.0125 were considered statistically significant.

**Results:**

Of 211 eligible patients, the frequencies of blood groups O, A, B, and AB were 89, 54, 56, and 12, respectively. Their respective months of median survival were 41, 20, 21, and 42. After adjustments in the six-and-twelve criteria and Child-Pugh scores, and using blood group O as the referent group, the coefficients (SE) of groups A, B, and AB were 0.69 (0.24), 0.47 (0.23), and 0.49 (0.49), respectively. A significant difference in survival was found only between patients with blood group O vs A (hazard ratio, 2.00; confidence interval, 1.25-3.21).

**Conclusions:**

ABO blood group is associated with the prognosis of HCC patients treated with TACE monotherapy. In our data, patients with blood group O tended to have the best survival. However, only blood group A patients had a significantly shorter survival rate comparing to blood group O.

## Introduction

Hepatocellular carcinoma (HCC) is a commonly diagnosed malignancy worldwide and the fourth leading cause of tumor-related deaths after lung, breast, and stomach cancers (Bray et al., 2018). The majority of HCC patients cannot undergo curative surgical resection due to multifocal disease, bilobar metastases, portal hypertension, or poor liver function. Transarterial chemoembolization (TACE) is the main standard treatment in patients with unresectable HCC, especially in Barcelona Clinic Liver Cancer (BCLC) stage B (intermediate stage) (Lo et al., 2002; Llovet et al., 2002; Llovet et al., 2008; Lencioni et al., 2016). The procedure precisely administers chemotherapeutic drugs emulsified with iodized oil to the tumor and blocks the tumor-feeding arteries with embolic agents. This results in combined ischemic and cytotoxic effects on the tumor cells. However, the median survival estimates of these unresectable HCC patients undergoing TACE is only 19.4 months (Lencioni et al., 2016). Additionally, unresectable-stage HCC is remarkably heterogeneous in terms of tumor size, number of nodules, and hepatic functional reserve (Kudo et al., 2019). Therefore, attempts to determine predictive factors are of great value to improve the surveillance of prognosis for HCC patients. Formerly, various factors were established to be prognostic indicators for HCC, such as advanced age, male gender, tumor size, high alpha-fetoprotein (AFP) level, and the presence of liver cirrhosis (Bannangkoon et al., 2018; Tirado et al., 2005). 

The ABO blood group is the most widely used erythrocyte antigen system in clinical practice and has an impact on the status of the host. Also, the ABO blood type is biologically correlated with many chronic diseases such as venous thromboembolism, preeclampsia, coronary heart disease, and carcinogenesis) (Tirado et al., 2005; Alpoim et al., 2013; He et al., 2012; Li et al., 2014). The association between ABO blood type and the risk of multiple malignancies has been reported and includes HCC) (Peng et al., 2016; Song et al., 2013; Engin et al., 2012; Zhou et al., 2015; Cao et al., 2014; Zhou et al., 2015; Iavarone et al., 2016; Shim et al., 2015). A previous study (Zhang et al., 2014) demonstrated that patients with blood group O have a significantly decreased risk of overall cancer compared with non-O groups. On the other hand, the same study reported that patients with blood group A have a significantly higher risk of cancer compared with non-A groups. However, only a few investigations (Wu et al., 2017; Li et al., 2018) have studied the prognostic role of ABO blood group in HCC patients following treatment. Therefore, for the first time, we evaluated the correlation between ABO blood group and the oncologic outcome of HCC patients who underwent TACE in the Thai population to bridge the aforementioned gap.

## Materials and Methods

*Patient population*

Approval to conduct this study was obtained from the institutional ethics committee of Songklanagarind Hospital which is a super-tertiary university hospital located on the campus of Prince of Songkla University in southern Thailand. We retrospectively reviewed medical records via the electronic hospital database (Health Information System) at our hospital of all HCC patients who underwent TACE between January 1, 2007 and December 31, 2019. For the diagnosis of HCC by imaging or histological findings, we followed the American Association for the Study of Liver Diseases criteria (Heimbach et al., 2018). We selected patients who met the following inclusion criteria: (a) HCC patients aged ≥18 years; (b) Child-Pugh score A5-B7; and (c) treated with TACE monotherapy. The excluded patients were those with impaired hepatic function (Child-Pugh class B8-9 and C), extrahepatic metastasis, infiltrative tumors, severe arterioportal shunt, history of spontaneous tumor rupture, cotreatment with any systemic or locoregional therapies during the TACE session, and the presence of vascular invasion.

All patients underwent investigations including complete blood count, liver function test, coagulation test, viral markers for hepatitis B and C infection, and serum AFP. The assessment of chronic liver disease was based on the Child-Pugh classification system. The laboratory data at the closest time prior to the first TACE were obtained. Baseline characteristics including age, sex, performance status, cause of HCC, and BCLC stage were also collected. Diagnoses of HCC and tumor burden, including tumor size and the number of nodules, were determined by radiologists and recorded in the Health Information System. 

Overall survival (OS) was defined as the length of time between the start date of treatment with TACE for liver cancer and the date of death or the last follow-up date. Patient status at the end of the study (August 31, 2020) was defined as alive or death using data from the Thailand civil registration database. Among 1,372 HCC patients in the database during the study period, a total of 211 patients who received TACE monotherapy due to HCC were eligible for the study. 

*Chemoembolization steps and techniques*

In this study, conventional TACE was defined as catheterization at the segmental hepatic artery performed by two interventional radiologists through the transfemoral route in all HCC patients. The celiac artery and superior mesenteric artery were selected at the beginning of the procedure. Subsequently, we performed selective catheterization to the tumor feeding hepatic arteries or in the extrahepatic collaterals that supplied each tumor using a microcatheter. After the microcatheter was inserted into the target arterial feeder, we slowly administered a mixture of iodized oil (range 4‒16 mL, Lipiodol, Guerbet, Villepinte, France) and doxorubicin hydrochloride (range 5‒50 mg, Adriamycin, Pfizer), or Vesimycin (range, 10-20 mg, Mitomycin-C, Naprod Life Sciences) under real-time monitoring with digital subtraction angiography (Allura Clarity FD20, Philips Healthcare, Best, the Netherlands). The amount of anticancer-in-oil emulsion was determined by total tumor size and the number of tumor nodules. The feeding artery was then embolized using gelatin sponge particles. Technical success was defined by successful catheter placement within the tumor-feeding branches and successful administration of chemoembolic agents to the target tumors. 

*Analysis of the prognostic factors for overall survival (OS)*

Univariate and multivariate analyses were used to determine the significant independent prognostic factors affecting OS. Nine clinical factors were analyzed: age, gender; Child-Pugh class; albumin-bilirubin (ALBI) score; etiology of HCC; liver cirrhosis; ascites; serum AFP level, and BCLC staging. Also analyzed were two tumor factors: the six-and-twelve criteria, defined as the sum of the size of the largest tumor in centimeters and the total number of tumor nodules, and multifocal tumor.

*Outcome and follow-up imaging studies*

All patients were followed up after conventional TACE with a detailed clinical examination, blood chemistries, and imaging examination (dynamic magnetic resonance imaging or a 4-phase contrast-enhanced computed tomography scan) one month after the initial procedure. If no definite evidence of residual tumor was demonstrated, imaging examinations were performed at 3- to 4-month intervals thereafter. A decision to repeat the TACE procedure was based on tumor response, BCLC stage of the HCC, and patient tolerance. 

*Statistical analysis*

Data analyses were performed using R software, version 3.2.2 (R foundation, Vienna, Austria). Numerical data are presented descriptively using the central tendency (mean, median, and mode) and a measure of dispersion (standard deviation and range). The cumulative OS rate was estimated using the Kaplan-Meier method and significant differences in survival distributions were tested by the log-rank test. The backgrounds of the patients were compared against their blood groups and survival analysis. The variables that affected OS (P <0.05) on univariate analysis were subsequently carried out using multivariate Cox’s regression model. Following Bonferroni adjustment for multiple comparisons of the blood groups, P-values <0.0125 were considered statistically significant. 

## Results

*Baseline characteristics of the patients*

The basic characteristics of the recruited patients classified by the ABO blood group are demonstrated in Table 1. This study enrolled 211 HCC patients (male, 69.2%) who underwent TACE monotherapy with a mean age of 60.7 ± 10.1 years (range, 35-84 years). The major etiology of HCC was hepatitis B virus (HBV) (48.8%), followed by hepatitis C virus (22.7%), and alcohol (11.8%). The Child-Pugh scores of liver cirrhosis were classified as A5 (41.7%), A6 (36.0%), and B7 (22.3%).

At the initial diagnosis of HCC, the mean tumor size was 4.5 ± 3.8 cm in diameter (range, 1.0-21.7 cm in diameter). About half of the patients (50.7%) had a multifocal tumor. According to the BCLC system, the percentages of cases classified as BCLC stage 0, A, and B were 10.0%, 54.0%, and 36.0%, respectively. 

The median values for baseline serum alanine aminotransferase and aspartate aminotransferase were 35 (10-138) U/L and 55 (18-259) U/L, respectively. The mean serum albumin level was 3.5 ± 0.6 g/dL. The median ALBI grading was 2 (1-3). Most patients had serum AFP levels ≤200 ng/mL (75.4%). The conventional TACE procedures achieved technical success in all 211 patients. The median number of TACE sessions was two per patient (1-10).

The frequencies of blood groups O, A, B, and AB were 89 (42.2%), 54 (25.6%), 56 (26.5%), and 12 (5.7%), respectively. The baseline characteristics of HCC patients were well balanced across the individual ABO blood group (Table 1).

*Factors associated with overall survival in the eligible patients*

Among the prognostic factors of interest, univariate analyses revealed that the Child-Pugh score (CP-A6 vs CP-A5, P = .006; CP-B7 vs CP-A5, P ≤ 0.001), serum AFP level ≤200 (P = 0.03), six-and-twelve criteria (score 6-12 vs ≤6, P ≤ 0.001; score >12 vs ≤6, P ≤ 0.001), BCLC staging (BCLC-A vs BCLC-0, P = 0.016; BCLC-B vs BCLC-0, P =0.004), and blood group (group A vs group O, P = 0.005; group B vs group O, P = 0.024; group non-O vs group O, P = 0.006) were the significant clinical prognostic factors predicting OS (Table 2). 

Subsequently, all five variables with P < 0.2 from univariate analyses were entered into the multivariable analysis. The results showed that the Child-Pugh score (CP-B7 vs CP-A5, P = 0.001), six-and-twelve criteria (score of >12 vs <6, P < 0.001), and ABO blood group (group A vs group O, P = .004; group non-O vs group O, P = 0.007) were the only three independently significant prognostic factors of OS after an adjustment in the multivariate analysis (Table 2). 

*Correlations of OS with ABO blood group*

Among the 211 study patients, there were 114 deaths (105 patients died from liver cancer and the rest of the patients died from other causes) after a median follow-up of 18 months (range, 1-162 months). The 1-, 2-, 3- and 5-year OS rates were 74.8%, 54.4%, 45.2%, and 31.4%, respectively. The median OS for the cohort was about 27.7 months (95% CI, 22.6-39.9 months). 

**Figure 1:**
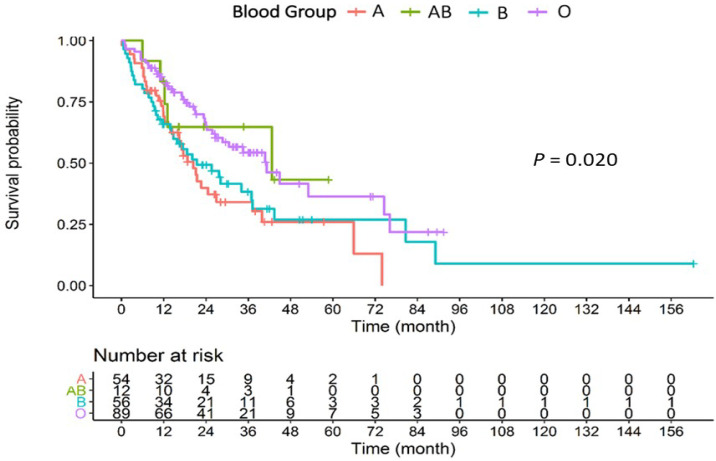
Survival Probability According to ABO Blood Groups

**Figure 2:**
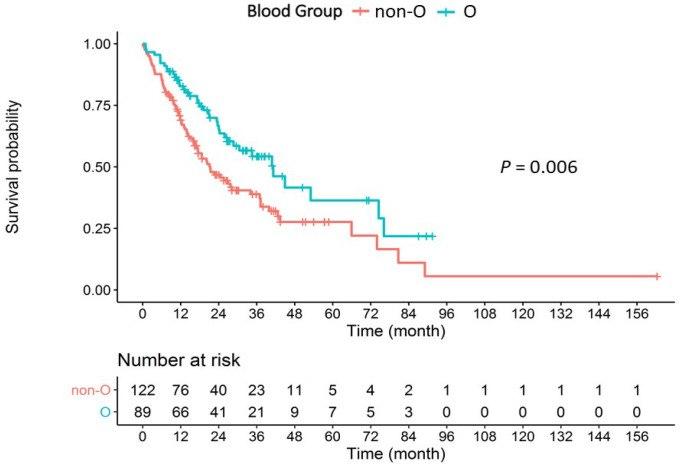
Survival Probability Compared between Blood Group O and Non-O

The median OS according to the patients’ blood group were: blood group O, 41.2 months (95% CI, 26.6-N/A months); blood group A, 20.3 months (95% CI, 16.3-37.2 months); blood group B, 21.4 months (95% CI, 14.7-37.2 months); and blood group AB, 42.6 months (95% CI, 13.0-N/A months). Using the log-rank test for the Kaplan-Meier survival analysis, there was a statistically significant difference in the effect of ABO blood group on OS (P = 0.02) (Figure 1). Additionally, the statistically significant difference on OS was demonstrated between patients with blood group O and those with non-O group (P = 0.006) (Figure 2).

After an adjustment in the multivariate analysis as mentioned earlier, using blood group O as the reference, the coefficients (standard error) of groups A, B, and AB were 0.70 (0.24), 0.39 (0.24), and 0.40 (0.50), respectively. After Bonferroni adjustment for multiple comparisons, P-values <.0125 were considered statistically significant. A significant difference in survival was found only between patients with blood group A vs O (hazard ratio, 2.00; 95% CI, 1.25-3.21; P = .004) (Table 3). 

**Table 1:** Characteristics of Patients Classified by ABO Blood Group

Variables, n (%)	Number	Blood group				P-value	Blood group		P-value
		O	A	B	AB		O	Non-O	
Total	211	89	54	56	12		89	122	
Age (years)						0.661			0.671
≥60	109	48 (53.9)	29 (53.7)	25 (44.6)	7 (58.3)		48 (53.9)	61 (50)	
<60	102	41 (46.1)	25 (46.3)	31 (55.4)	5 (41.7)		41 (46.1)	61 (50)	
Sex						0.574			0.744
Male	146	60 (67.4)	41 (75.9)	38 (67.9)	7 (58.3)		60 (67.4)	86 (70.5)	
Female	65	29 (32.6)	13 (24.1)	18 (32.1)	5 (41.7)		29 (32.6)	36 (29.5)	
Child-Pugh score						0.456			0.372
A5	88	41 (46.1)	22 (40.7)	18 (32.1)	7 (58.3)		41 (46.1)	47 (38.5)	
A6	76	32 (36)	17 (31.5)	24 (42.9)	3 (25)		32 (36)	44 (36.1)	
B7	47	16 (18)	15 (27.8)	14 (25)	2 (16.7)		16 (18)	31 (25.4)	
ALBI score						0.699			0.448
1	77	33 (37.1)	18 (33.3)	20 (35.7)	6 (50)		33 (37.1)	44 (36.1)	
2	119	52 (58.4)	30 (55.6)	32 (57.1)	5 (41.7)		52 (58.4)	67 (54.9)	
3	15	4 (4.5)	6 (11.1)	4 (7.1)	1 (8.3)		4 (4.5)	11 (9)	
Etiology						0.153			0.045
HBV	103	41 (46.1)	29 (53.7)	29 (51.8)	4 (33.3)		62 (50.8)	41 (46.1)	
HCV	48	15 (16.9)	12 (22.2)	15 (26.8)	6 (50)		33 (27)	15 (16.9)	
Alcohol	25	16 (18)	4 (7.4)	5 (8.9)	0 ())		9 (7.4)	16 (18)	
Others	35	17 (19.1)	9 (16.7)	7 (12.5)	2 (16.7)		18 (14.8)	17 (19.1)	
Liver cirrhosis						0.527			1.000
Yes	202	85 (95.5)	53 (98.1)	53 (94.6)	11 (91.7)		85 (95.5)	117 (95.9)	
No	9	4 (4.5)	1 (1.9)	3 (5.4)	1 (8.3)		4 (4.5)	5 (4.1)	
Ascites						0.138			0.438
Yes	32	11 (12.4)	8 (14.8)	13 (23.2)	0 (0)		11 (12.4)	21 (17.2)	
No	179	78 (87.6)	46 (85.2)	43 (76.8)	12 (100)		78 (87.6)	101 (82.8)	
Serum AFP						0.302			0.431
>200	52	19 (21.3)	11 (20.4)	19 (33.9)	3 (25)		19 (21.3)	33 (27)	
≤200	159	70 (78.7)	43 (79.6)	37 (66.1)	9 (75)		70 (78.7)	89 (73)	
Six-and-twelve criteria						0.138			0.232
Score ≥6		48 (53.9)	33 (61.1)	29 (51.8)	11 (91.7)		48 (53.9)	73 (59.8)	
Score 6-12		28 (31.5)	18 (33.3)	21 (37.5)	1 (8.3)		28 (31.5)	40 (32.8)	
Score >12		13 (14.6)	3 (5.6)	6 (10.7)	0 (0)		13 (14.6)	9 (7.4)	
Multifocal tumor						0.996			0.918
Yes	107	46 (51.7)	27 (50)	28 (50)	6 (50)		46 (48.3)	61 (50)	
No	104	43 (48.3)	27 (50)	28 (50)	6 (50)		43 (48.3)	61 (50)	
BCLC staging						0.870			0.908
BCLC-0	21	8 (9)	5 (9.3)	6 (10.7)	2 (16.7)		8 (9)	13 (10.7)	
BCLC-A	114	48 (53.9)	32 (59.3)	27 (48.2)	7 (58.3)		48 (53.9)	66 (54.1)	
BCLC-B	76	33 (37.1)	17 (31.5)	23 (41.1)	3 (25)		33 (37.1)	43 (35.2)	

Values are presented as number (%). Abbreviations: ALBI, albumin-bilirubin; HBV, hepatitis B virus; HCV, hepatitis C virus; AFP, alpha-fetoprotein; BCLC, Barcelona Clinic Liver Cancer.

**Table 2:** Cox Regression Analysis of Potential Prognostic Factors

Variables	Univariate analysis		Multivariate analysis	
	HR (95% CI)	P-value	HR (95% CI)	P-value
Age (years)				
≥60 vs <60	0.91 (0.63-1.32)	0.625		
Sex				
Male vs Female	0.93 (0.63-1.38)	0.72		
Child-Pugh score				
A6 vs A5	1.83 (1.19-2.81)	0.006	1.63 (1.04-2.56)	0.035
B7 vs A5	2.82 (1.71-4.65)	<.001	2.31 (1.38-3.86)	0.001
ALBI score				
2 vs 1	1.67 (1.12-2.49)	0.012		
3 vs 1	2.23 (1.07-4.65)	0.033		
Etiology				
HBV vs Others	0.88 (0.52-1.47)	0.614		
HCV vs Others	1.21 (0.68-2.16)	0.522		
Alcohol vs Others	1.31 (0.67-2.56)	0.433		
Liver cirrhosis				
Yes vs No	0.5 (0.23-1.08)	0.109		
Ascites				
Yes vs No	1.93 (1.20-3.09)	0.011		
Serum AFP				
>200 vs ≤200	1.91 (1.28-2.86)	0.003		
Six-and-twelve criteria				
Score >6-12 vs ≤6	2.09 (1.40-3.12)	<.001	1.90 (1.26-2.87)	0.002
Score >12 vs ≤6	4.41 (2.48-7.85)	<.001	3.88 (2.13-7.10)	<0.001
Multifocal tumor				
Yes vs No	1.2 (0.83-1.73)	0.333		
BCLC staging				
BCLC-A vs BCLC-0	2.8 (1.21-6.49)	0.016		
BCLC-B vs BCLC-0	3.48 (1.48-8.18)	0.004		
Blood group				
A vs O	1.94 (1.22-3.08)	0.005	2.00 (1.25-3.21)	0.004
B vs O	1.69 (1.07-2.66)	0.024	1.53 (0.97-2.41)	0.070
AB vs O	1.05 (0.41-2.66)	0.926	1.65 (0.63-4.30)	0.308
Non-O vs O	1.72 (1.16-2.53)	0.006	1.71 (1.16-2.53)	0.007

Abbreviations: ALBI, albumin-bilirubin; HBV, hepatitis B virus; HCV, hepatitis C virus; AFP, alpha-fetoprotein; BCLC, Barcelona Clinic Liver Cancer

**Table 3:** Comparison of P values among the Four Blood Groups. Right upper cells are from univariate analysis and left lower cells are from multivariate analysis

	A	B	AB	O
A		0.568	0.198	0.005*
B	0.356		0.318	0.024
AB	0.682	0.961		0.926
O	0.004*	0.07	0.308	

## Discussion

HCC is an aggressive tumor and the fourth leading cause of tumor-related deaths worldwide (Bray et al., 2018). HBV infections are a major global public health problem (Zhou et al., 2015) and are the most common cause of liver tumor as also demonstrated in our study. Many studies demonstrated that advanced age, male gender, tumor size, high AFP level, and the presence of liver cirrhosis are established prognostic factors for HCC. (Fattovich et al., 2004; Bannangkoon et al., 2018)

 In our study, the three significant prognostic factors that affected the survival of HCC patients who underwent TACE were the ABO blood type, Child-Pugh score, and six-and-twelve criteria. Our cohort showed that ABO blood group was associated with OS in a study of HCC patients who underwent TACE monotherapy. The prognosis and overall outcome were worse in HCC patients with blood group A compared with blood group O. Our results also demonstrated that patients with blood group O tended to have the best overall survival. The results of our study were similar to previous reports (Wu et al., 2017; Li et al., 2018) that demonstrated a better prognosis and oncologic outcome in HCC patients with blood type O compared with non-O groups. However, there were some differences in terms of methodology. In previous reports (Wu et al., 2017; Li et al., 2018), the evaluation of the prognostic role of ABO blood group was performed in HCC patients who underwent hepatectomy or initial treatment with TACE. In our cohort, we enrolled BCLC stage 0/A and B HCC patients treated with TACE monotherapy. TACE is the most common mode of treatment in Thailand (Bannangkoon et al., 2018) and the prognosis of patients treated with selective TACE is not inferior to those who treated with ablation in some patients who are ineligible for ablation therapy (Ishikawa et al., 2018). 

The mechanisms of ABO blood group affecting the oncologic outcome of HCC patients are still controversial. Two studies Okada et al., (1987) and Okada et al., (1987) reported the ABO antigen has been documented as a cancer-associated antigen. The expression of blood group antigens was found not only on the human hepatocellular cancer cell surface but also in the adjacent non-tumorous liver tissues in laboratory results. These findings indicated a prognostic role of the ABO blood group antigens in the malignant transformation of hepatocytes. Additionally, a strong correlation between plasma alkaline phosphatase and genetic polymorphisms within the ABO locus on chromosome 9q34.13 (Reid et al., 2004) represented a link between ABO blood group and liver function (Yuan et al., 2008). The blood group O is classified by the presence of the H antigen and the anti-A and anti-B antibodies in the serum (natural isoagglutinins) which could perform as a cancer defense factor (Reid et al., 2004; Yamamoto et al., 2012). Moreover, the half-life of von Willebrand factor in patients with blood group O is significantly shorter than in the non-O group which was shown to result in a lower risk of venous thromboembolism in patients with blood group O (Jenkins et al., 2006; Gallinaro et al., 2008). Also, hypercoagulability associated with the potential of metastasis resulted in a low response to treatment and poor survival (Tsimafeyeu et al., 2009). Thus, the non-O group patients have a greater propensity of hypercoagulability and hypervascularity which are typical aggressive HCC characteristics. In addition, selectins and cellular adhesion molecules (CAMs), that contribute to inflammatory processes by stimulating adhesion of leukocytes to the vascular wall endothelium, has been linked with metastasis potential and tumor progression (Kobayashi et al., 2007; Barbalic et al., 2010). Patients with blood group O, who have higher level of selectins and CAMs compared to those with non-O group, may have a lower risk of tumor spreading (Larson et al., 2016). The aforementioned laboratory findings may explain the apparent correlation between ABO blood type and the oncologic outcome in HCC patients. 

The Child-Pugh scoring system is the most widely used method for evaluation of liver function and plays an important role as a staging system to guide treatment in HCC patients (Bruix et al., 2011; EASL-EORTC, 2012). Additionally, the Child-Pugh classification is also used to predict therapeutic effectiveness. The key issue when predicting the treatment outcome of HCC patients is to assess the liver function reserve to select proper candidates for TACE. In several studies, TACE has primarily proved to improve survival in HCC; however, most of the studies included Child A patients (Llovet et al., 2003; Camma et al., 2002). Currently, TACE is recommended for Child A and highly selected Child B by the American Association for the Study of Liver Diseases (Heimbach et al., 2018). The Thai guideline for the management of HCC (2019) recommends that a Child-Pugh score ≥9 is an absolute contraindication for TACE (THASL, 2019). However, the applicability of the Child-Pugh system has some limitations. The evaluation of encephalopathy and ascites is subjective, and the Child-Pugh classification only addresses the liver functional capacity without including any tumor factors. 

The six-and-twelve criteria was specifically developed to predict the prognosis in HCC patients undergoing TACE and has been recently validated (Wang et al., 2019; Kaewdech et al., 2021). The present study showed that, even after an adjustment with the six-and-twelve criteria which were developed explicitly for such patients undergoing TACE monotherapy, ABO blood group, especially blood group A, remained an independent prognostic factor for a poorer survival in HCC patients. 

To our knowledge, this is the first study to confirm the association between ABO blood type and oncologic outcome of HCC patients who underwent TACE monotherapy. We also recognize some limitations of our study. First, this study was limited by its retrospective nature at a single center. Second, the number of patients in this series was relatively small due to a quite narrow window of the study population. Third, the ABO blood group is related to ethnicity, but all HCC patients were ethnic Thai. Finally, the limited number of patients with blood group AB may be the confounder to interpret the survival outcome. 

In conclusion, ABO blood group is associated with the prognosis of HCC patients treated with TACE monotherapy. HCC patients with blood group O tended to have the best survival. However, only blood group A patients had significantly shorter survival. Further studies are needed to determine how ABO blood groups may influence cancer development and progression after treatment and to develop risk prediction models in patients with cancer. Additionally, the pattern of treatment failure in patients with blood group O compared to those with non-O group should be evaluated.

## Author Contribution Statement

Kittipitch Bannangkoon, MD: Study concept and design, Data acquisition, Data analysis and interpretation, Drafting manuscript, Critical revision of manuscript for important intellectual content, Statistical analysis, Study supervision. Keerati Hongsakul, MD: Study concept and design, Data acquisition, Data analysis and interpretation, Drafting manuscript, Statistical analysis, Study supervision. Pimsiri Sripongpun, M.D.: Study concept and design, Data acquisition, Data analysis and interpretation, Drafting manuscript, Statistical analysis, Study supervision. Apichat Kaewdech, M.D.: Study concept and design, Data acquisition, Data analysis and interpretation, Drafting manuscript. Naichaya Chamroonkul, M.D.: Study concept and design, Data analysis and interpretation, Drafting manuscript. Teeravut Tubtawee, M.D.: Study concept and design, Data acquisition, Drafting manuscript. Teerha Piratvisuth, M.D.Study concept and design, Study supervision

## Ethical Approval

The study was approved by the Ethics Committee of Faculty of Medicine, Prince of Songkla University (64-088-7-1). 

## Conflict of interest disclosure

The authors have no competing interests to declare.
